# Alkali modified P25 with enhanced CO_2_ adsorption for CO_2_ photoreduction

**DOI:** 10.1039/d0ra05010e

**Published:** 2020-07-27

**Authors:** Jeannie Z. Y. Tan, Stelios Gavrielides, Hao R. Xu, Warren A. Thompson, M. Mercedes Maroto-Valer

**Affiliations:** Research Centre for Carbon Solutions (RCCS), Heriot-Watt University Edinburgh EH14 4AS UK j.tan@hw.ac.uk; Department of Chemical Engineering, Loughborough University Loughborough UK

## Abstract

To improve the CO_2_ adsorption on the photocatalyst, which is an essential step for CO_2_ photoreduction, solid solutions were fabricated using a facile calcination treatment at 900 °C. Using various alkalis, namely NaOH, Na_2_CO_3_, KOH, K_2_CO_3_, the resulted samples presented a much higher CO_2_ adsorption capacity, which was measured with the pulse injection of CO_2_ on the temperature programmed desorption workstation, compared to the pristine Evonik P25. As a result, all of the fabricated solid solutions produced higer yield of CO under UV light irradiation due to the increased basicity of the solid solutions even though they possessed only the rutile polymorph of TiO_2_. The highest CO_2_ adsorption capacity under UV irradiation was observed in the sample treated with NaOH, which contained the highest amount of isolated hydroxyls, as shown in the FTIR studies.

## Abstract 2

Enhanced CO_2_ adsorption capability and photocurrent with alkali modified P25 for CO_2_ photoreduction.

## Graphical abstract

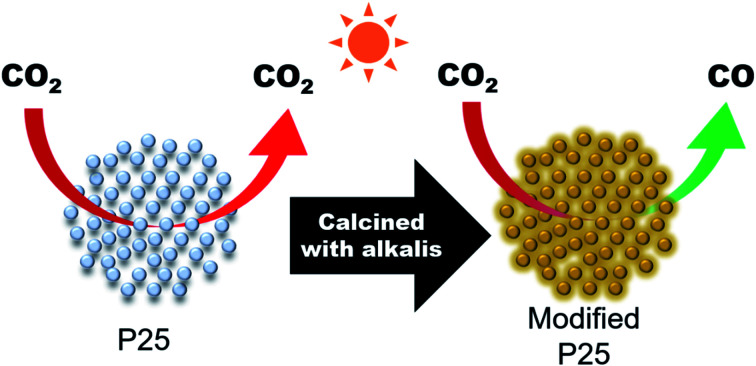

## Introduction

The utilisation of fossil fuels and associated greenhouse effect have raised lots of concerns. Solar energy has the potential to meet our energy demands if it can be efficiently harvested and transformed into fuels. Photocatalytic reduction of CO_2_ into valuable hydrocarbons, such as CO and CH_4_, is promising to reduce CO_2_ emissions by offering a renewable energy alternative.^1^

Photocatalytic reduction of CO_2_ involves multiple reaction steps before producing the hydrocarbon products.^2,3^ However, as the most abundant and stable oxidised form of carbon, CO_2_ is difficult to activate.^4^ Surface chemistry studies have demonstrated that adsorption of CO_2_ molecules on the surface of metal oxides is always accompanied by an activation step.^5^ CO_2_ in the chemisorption state (mainly carbonate and/or CO_2_^−^ anion) has a bent O–C–O bond angle and a decreased LUMO, which will favour the charge transfer from photo-excited semiconductors to the surface adsorbed CO_2_ molecules.^6,7^

Adsorption of CO_2_ on the surface of the photocatalyst is known as the first essential step in a CO_2_ photoreduction reaction. However, the competition between CO_2_ and H_2_O (*i.e*., reducing agent) for the adsorption sites on the surface of the photocatalyst could significantly affect the CO_2_ photoreduction efficiency. To understand the effects of the strength and state of CO_2_ adsorption on photocatalytic CO_2_ reduction, various methods have been proposed to enhance the adsorption of CO_2_ as well as to enhance the chemical interaction of CO_2_ with the photocatalyst. One simple way is through the addition of alkalis and alkaline earth metals, such as NaOH and Na_2_CO_3_,^8^ in order to enhance the adsorption capacity^9^ and activation of CO_2_ (ref. 5 and 10) without the use of noble metals, such as Pt.^5^ Nie *et al.* proposed that the modification of P25 with NaOH to introduce more surface hydroxyl groups that would establish strong hydrogen bonding on TiO_2_, and subsequently promote the electron transfer between the reactant and TiO_2_.^9^ As a result, the photocatalytic conversion of the NaOH modified P25 titania was improved approximately 12 times when compared to the pristine P25 photocatalyst.

A better understanding of the surface hydroxyls of TiO_2_, especially their role in the photocatalytic process, may provide insight into the photocatalytic mechanism. A previous study proposed that the type of hydroxyl group within TiO_2_ could significantly influence the photocatalytic reaction due to the difference in acidic–basic strength of the hydroxyl group.^11^ Liu *et al.* recently proposed that isolated hydroxyls could act as an effective adsorption site for CO_2_ as well as enhanced the selectivity for the production of hydrocarbon fuels from CO_2_ and H_2_O.^12^ Using infrared spectroscopy, the bands observed between 3715–3630 and 3675 cm^−1^ were assigned to the stretching modes of isolated –OH, whereas bands at lower frequencies of 1640–1625 cm^−1^ were assigned to the bending modes of adsorbed water.^13–15^ The isolated –OH was categorised into terminal and bridged –OH groups. Some studies also indicated that the preparation method, which produced different size and morphology of particles, had a great effect on the –OH frequencies.^16,17^ Moreover, UV irradiation would lead to the changes of the concentration and structure of the hydroxyl groups on TiO_2_ surface.^18,19^ The behaviour of the surface –OH as source of hydroxyl radicals as well as the adsorption sites for the reactant in the photocatalytic processes has been described.^13^ However, the relationship between the surface –OH and the capability of CO_2_ adsorption under light irradiation is not fully understood.

Surface modification with alkalis has shown advantages for CO_2_ photocatalytic reduction. However, the influence of anionic and cationic moieties of the alkalis on CO_2_ adsorption capacity and the CO_2_ photoreduction activity has not been systematically studied. Therefore, this study aims to understand the influence of commonly used alkalis, namely NaOH, Na_2_CO_3_, KOH, K_2_CO_3_, in modifying commercial TiO_2_, P25 photocatalyst. The effect of anions and cations on the CO_2_ adsorption capability and CO_2_ photoreduction under UV is investigated here using temperature programmed desorption analysis.

## Experimental

### Materials

Aeroxide® P25 (≥99.5%), referred here to as P25 in short, NaOH (BioXtra, ≥98.0%), Na_2_CO_3_ (ACS reagent, ≥99.5%), KOH (BioXtra, ≥85.0%) and K_2_CO_3_ (BioXtra, ≥99.0%) were purchased from Sigma Aldrich. All reagents were used without further purification. Milli-Q water was collected from a Millipore academic purification system with resistivity 18.2 MΩ cm.

### Methods

P25 (0.1 g) was mixed and ground thoroughly with 5 wt% of NaOH, Na_2_CO_3_, KOH or K_2_CO_3_. The samples were annealed at 900 °C (ramping rate: 10 °C min^−1^) for 4 h and they were denoted as P25-X, in which X = NO, NC, KO and KC represents the NaOH, Na_2_CO_3_, KOH and K_2_CO_3_, respectively. For comparison, P25 was also calcined under the same conditions and this sample was denoted as P25-C.

### Characterisation

Crystallinity and phase identification of the synthesized samples were conducted using powder X-ray diffraction XRD (Bruker D8 Advanced Diffractormeter) equipped with Cu Kα radiation (*λ* = 1.5418 Å) and compared with the ICDD–JCPDS Powder Diffraction File database. Transient photocurrent response was measured using Autolab PGSTAT 302N electrochemical workstation with a standard three-electrode system, in which fabricated thin film acted as the working electrode (2.5 × 2.5 cm^2^), Pt wire as counter electrode and Ag/AgCl (KCl 1 M) as reference electrode, and 0.1 M Na_2_CO_3_ aqueous solution was used as the electrolyte. Zeta potential was measured using a Zetasizer Nano Z from Malvern Panalytical. Each sample was measured with 4 replicas. The measurement was conducted with the fabricated samples diluted in Milli-Q water (0.167 mg mL^−1^) at 22 °C. Fourier transform infrared (FTIR) spectroscopy (Frontier from PerkinElmer) was used to obtain the fingerprint of the powder samples. Surface area was measured using Gemini VII Surface Area Analyser (Micromeritics). Temperature Programmed Desorption (TPD, ChemBET Pulsar TPD from Quantachrome Instruments) studies were performed using the pulse injection method to measure the volume of CO_2_ adsorbed under UV irradiation (OmniCure S2000, 75 mW cm^−1^, 365 nm) at 25 °C. Prior to the measurement, 0.01 g of sample was filled into a U-shape quartz tube and degassed at 150 °C for 4 h. After cooling down to 25 °C, the quartz tube was filled with CO_2_ (99.99%) and the measurement was proceeded for 5500 s under UV irradiation. Then, the quartz tube was purged with CO_2_ for 24 h before the next measurement for another 4500 s. To estimate the amount of CO_2_ adsorbed, the area under the curve obtained in each injection was compared with the area under the curve obtained when an empty (no sample loaded in the quartz tube) was used in the injection of CO_2_.

### Photocatalytic testing

CO_2_ photoreduction testing of the produced samples was conducted in a customised stainless steel photoreactor with a quartz window.^20^ 0.01 g of sample was distributed as powder on the bottom of the photoreactor. To purge and equilibrate the system, a flow rate of 0.42 mL min^−1^ CO_2_ was passed through an aluminium bubbler set at 20 ± 2 °C and charged the photoreactor overnight. The reaction was performed at 24 ± 2 °C. An optical fibre lamp (OmniCure S2000) was used as the light source (75 mW cm^−1^, 365 nm). The irradiance was measured using a radiometer (OmniCure R2000). The outlet gas was analysed hourly online by a gas chromatography (GC, Agilent, Model 7890 B series) with a Hayesep Q column (1.5 m, 1/16 inch OD, 1 mm ID), molecular sieve 13X (1.2 m, 1/16 inch OD, 1 mm ID), thermal conductivity detector (TCD), nickel catalysed methaniser and flame-ionization detector (FID).

The quantum yield (*ϕ*) was measured under similar photocatalytic reaction conditions using the same UV lamp (75 mW cm^−1^, 365 nm). The incident flux was determined by a Laboratory Spectroradiometer (Apogee Instruments). The *ϕ* values of CO evolution for the CO_2_ photoreduction reaction were calculated according to the following equation:
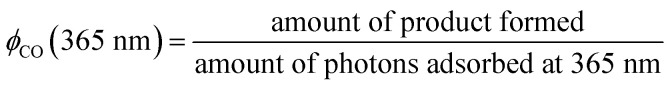


## Results and discussion

**Fig. 1:**
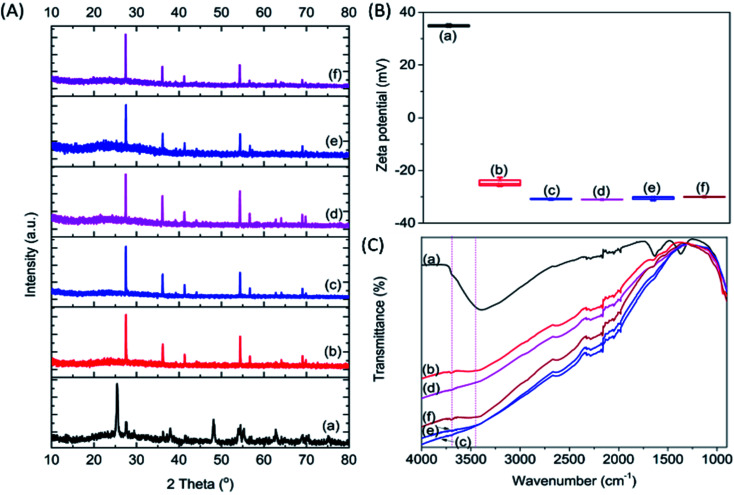
XRD (A), zeta potential (B) and FTIR (C) patterns of P25 (a) P25-C (b) P25-NO (c) P25-NC (d) P25-KO (e) and P25-KC (f).

Four types of alkalis, namely NaOH, Na_2_CO_3_, KOH and K_2_CO_3_, were used to modify the commercial P25, which has been extensively used as a benchmark photocatalyst.^21^ To distinguish the effect of high temperature calcination and salt modification, pristine P25 was treated at 900 °C without the addition of salt. The modified P25, and resultant P25-C, P25-NO, P25-NC, P25-KO and P25-KC, revealed 100% rutile crystal phase, whereas pristine P25 had a mixture of anatase and rutile (Fig. 1A). This observation indicated that the phase transformation was primarily due to the calcination treatment.^22^

The surface area of the samples after the calcination treatment with (46.4–48.3 m^2^ g^−1^) and without (48.5 m^2^ g^−1^) the incorporation of alkali was reduced slightly compared to the pristine P25 (51.7 m^2^ g^−1^). However, the calcination treatment reduced the zeta potential value significantly from 35 mV (pristine P25) to −24.5 mV (P25-C). The addition of salts further decreased the zeta potential value to about −32 mV (Fig. 1B). This was due to the increase of the basicity of the materials upon the addition of alkalis. However, the type of salt added did not significantly affect the zeta potential.

Pristine P25 revealed a strong trough centred at 3391 cm^−1^, which was assigned to the O–H stretching vibration bands, due to water adsorption (Fig. 1C).^23^ Upon calcination, this trough was weakened and this was also the case of the trough at 1630 cm^−1^, which was assigned to the bending modes of adsorbed water,^24,25^ indicating the dehydration of the sample.^11^ In addition to the decline of troughs, a very broad band emerged between 3600–3800 cm^−1^ due to the formation of Ti^3+^–OH and/or Ti^4+^–OH moieties (*i.e.*, isolated hydroxyl groups) after the addition of alkalis as proposed previously.^26^

The calcination treatment with or without the incorporation of alkalis with different anions (*i.e.*, –OH^−^ and –CO_3_^2−^) and cations (*i.e.*, Na^+^ and K^+^) changed the basicity, resulting from the formation of different amounts of isolated –OH groups in the modified P25. The amount of isolated –OH formed after calcination within the samples decreased in the order of P25-NO > P25-KO > P25-NC > P25-KC > P25-C (Fig. 1C). Therefore, these observations proposed that hydroxides could induce more isolated –OH groups than carbonates during the alkali treatment.

**Fig. 2:**
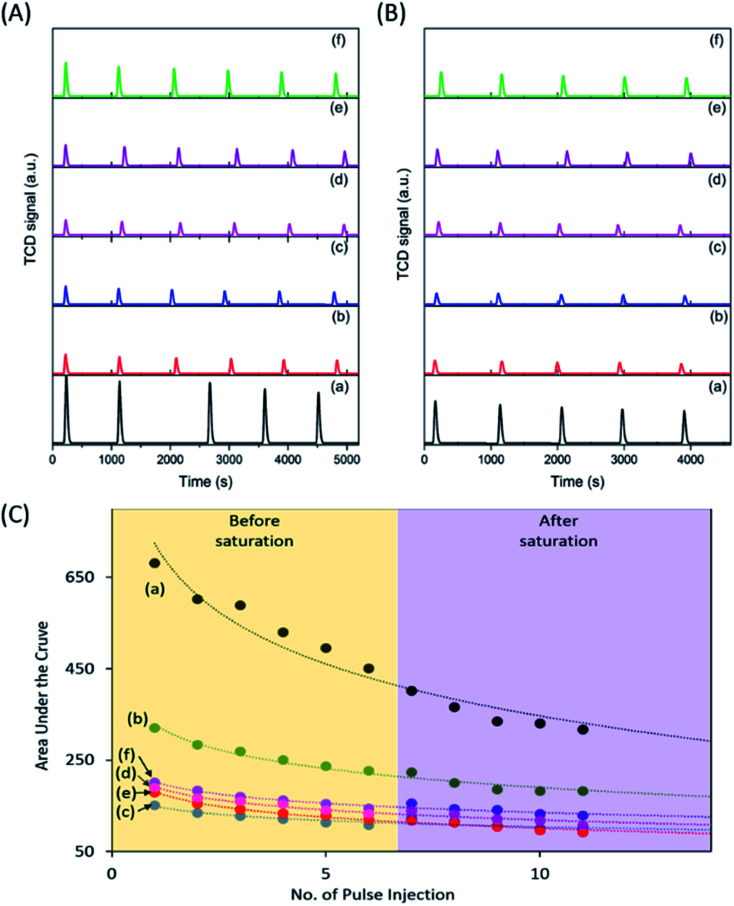
Pulse injection of CO_2_ performed on TPD before (A) and after (B) CO_2_ equilibrated for 24 h, and the integrated area under the curve (C) for pristine P25 (a) P25-C (b) P25-NO (c) P25-NC (d) P25-KO (e) and P25-KC (f) under UV irradiation.

To investigate the CO_2_ adsorption capacity of the samples, pulse injection of CO_2_ under UV light irradiation studies were performed using a TPD instrument (Fig. 2A and B). The pulse injection pattern showed that pristine P25 exhibited the highest signal amongst the samples, indicating the least amount of CO_2_ injected into the sample. However, all calcined samples exhibited a much lower signal compared to pristine P25, indicating a much higher amount of CO_2_ adsorbed by those calcined samples. The area under the curve was determined to estimate the volume of CO_2_ adsorbed (Fig. 2C). The area under the curve reached a plateau after 6 injections, indicating that the samples had been saturated with CO_2_ after being equilibrated with CO_2_ for 24 h (purple region in Fig. 2C). Based on the integration of the graphs obtained, the area under the curve increased in the order of P25-NO < P25-KO < P25-NC < P25-KC < P25-C (Fig. 2C). However, the estimated volume of CO_2_ adsorbed by the samples had an inverse relation to the area under the curve (Table 1). In other words, the capability of CO_2_ adsorption decreased in the order of P25-NO > P25-KO > P25-NC > P25-KC > P25-C, which revealed similar trend as shown in the FTIR pattern in Fig. 1C. In other words, the amount of isolated –OH present in the samples directly influenced the CO_2_ adsorption capacity, as previously proposed.^26^

**Table 1:** Integrated area under the curve and the corresponding CO_2_ volume adsorbed estimated after 11 injections of CO_2_

Sample	Area under the curve	Estimated CO_2_ volume adsorbed (mL g^−1^)
P25	316.4	16.3
P25-C	181.8	20.0
P25-NO	92.1	22.4
P25-NC	106.7	21.9
P25-KO	99.6	22.2
P25-KC	128.9	21.4

**Fig. 3:**
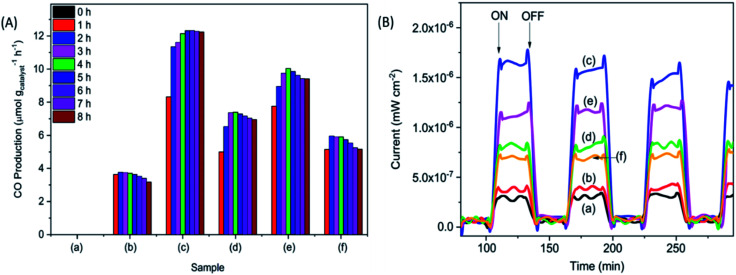
CO production (A) and photocurrent (B) obtained from for the pristine P25 (a) P25-C (b) P25-NO (c) P25-NC (d) P25-KO (e) and P25-KC (f) under UV irradiation.

The photocatalytic reduction of CO_2_ was performed under UV irradiation for 8 h (Fig. 3A). No carbonaceous product was observed when the samples were tested in the dark. The pristine P25 sample did not produce observable products, whereas, the calcined samples produced CO under UV irradiation. The CO production decreased in the order of P25-NO > P25-KO > P25-NC > P25-KC > P25-C. The higher CO production was observed for the P25 treated with hydroxides, namely P25-NO produced 12.3 μmol g^−1^ h^−1^ with a *ϕ*_CO_ = 0.0167, followed by P25-KO (10.1 μmol g^−1^ h^−1^). Samples treated with carbonates presented lower conversion, *e.g.* 7.4 and 5.9 μmol g^−1^ h^−1^ of CO by P25-NC and P25-KC, respectively.

Transient photocurrent response is used to reveal the migration characteristics of photogenerated electrons.^27^ Overall, the samples treated with alkalis exhibited higher photocurrent than the pristine P25 and P25-C samples (Fig. 3B). Among the modified P25 samples, P25-NO revealed the highest photocurrent, indicating the resulted P25-NO solid solution had enhanced the transportation of the photogenerated electrons, that subsequently improved the photocatalytic conversion of CO_2_ into CO.^27^ The superior electronic property of P25-NO was probably due to higher relative amount of Ti^3+^–OH/Ti^4+^–OH formation than P25-KO. Hence, P25-NO revealed a much higher photocatalytic activity than P25-KO although they revealed very small difference in their CO_2_ adsorption capacities (Table 1).

In summary, the sample capable to adsorb the highest amount of CO_2_ (Fig. 2) exhibited highest isolated –OH concentration, as shown in the FTIR pattern (Fig. 1C). In addition, the modification of P25 with alkalis generally enhanced the transport of photogenerated electrons, as shown in the photocurrent analysis (Fig. 3B). Among the modified samples, P25-NO revealed the highest photocurrent. The synergistic effects of the enhanced CO_2_ adsorption and the transportation of photogenerated electrons had significantly promoted the photoreduction of CO_2_ into CO under UV irradiation.

## Conclusions

The modification of P25 with different alkalis showed significant CO_2_ enhancement attributed to the increase of surface basicity as well as isolated hydroxyls. In addition, the fabricated solid solutions could produce more photogenerated electrons and transported effectively to the surface for CO_2_ photoreduction. As a result, the fabricated solid solutions exhibited much higher CO production from CO_2_ under UV light irradiation. The sample treated with NaOH exhibited the highest CO_2_ adsorption capacity (∼22.4 mL g^−1^) and produced ∼13 μmol g_catalyst_^−1^ h^−1^ of CO from the CO_2_ photoreduction reaction with H_2_O under UV irradiation.

## Conflicts of interest

There are no conflicts to declare.

## Supplementary Material
